# Improved Pre-miRNA Classification by Reducing the Effect of Class Imbalance

**DOI:** 10.1155/2015/960108

**Published:** 2015-11-10

**Authors:** Yingli Zhong, Ping Xuan, Ke Han, Weiping Zhang, Jianzhong Li

**Affiliations:** ^1^School of Computer Science and Technology, Key Laboratory of Database and Parallel Computing of Heilongjiang Province, Heilongjiang University, Harbin 150080, China; ^2^School of Computer and Information Engineering, Harbin University of Commerce, Harbin 150028, China

## Abstract

MicroRNAs (miRNAs) play important roles in the diverse biological processes of animals and plants. Although the prediction methods based on machine learning can identify nonhomologous and species-specific miRNAs, they suffered from severe class imbalance on real and pseudo pre-miRNAs. We propose a pre-miRNA classification method based on cost-sensitive ensemble learning and refer to it as MiRNAClassify. Through a series of iterations, the information of all the positive and negative samples is completely exploited. In each iteration, a new classification instance is trained by the equal number of positive and negative samples. In this way, the negative effect of class imbalance is efficiently relieved. The new instance primarily focuses on those samples that are easy to be misclassified. In addition, the positive samples are assigned higher cost weight than the negative samples. MiRNAClassify is compared with several state-of-the-art methods and some well-known classification models by testing the datasets about human, animal, and plant. The result of cross validation indicates that MiRNAClassify significantly outperforms other methods and models. In addition, the newly added pre-miRNAs are used to further evaluate the ability of these methods to discover novel pre-miRNAs. MiRNAClassify still achieves consistently superior performance and can discover more pre-miRNAs.

## 1. Introduction

MicroRNAs (miRNAs) are a set of short noncoding RNAs (~22 nt) which bind with the mRNAs to regulate their expression and result in their cleavage or translational repression [1, 2]. The miRNAs usually participate in plenty of biological processes of animals and plants, including the developmental process, hematopoietic process, organogenesis, cell apoptosis, and cell proliferation in animals as well as growth and signal transduction in plants [3, 4]. An effective route for identifying miRNA is to predict the candidates by using computational methods, following by purposeful validation with the biological experiments.

For the computational prediction of miRNAs, the methods based on homologous search, comparative genomics, and machine learning are three major categories. Although the first two kinds of methods can accurately identify miRNAs, they cannot identify those nonhomologous and species-specific miRNAs. The main reason is that they depend on sequence homology and sequence conservation among the multiple species. The prediction based on machine learning is a* de novo* method which can identify nonhomologous and species-specific miRNAs. A prediction method based on machine learning uses real pre-miRNAs and pseudo pre-miRNAs as positive samples and negative samples, respectively. Many features are extracted from these samples according to the sequence and structure characteristics of pre-miRNAs. At last, a classification model is trained by using the samples and their features. So far, researchers have established models based on naive Bayes [5, 6], support vector machine (SVM) [7–9], Random Forest [10], and probabilistic colearning [11] and used these models to discriminate whether a given new sequence is a real pre-miRNA. Those sequences classified as real pre-miRNAs can be regarded as candidates. The position prediction method [12–14] is used to further determine the locations of miRNAs in the candidates. All pre-miRNA candidates and the location information of their miRNAs contribute to the subsequent biological validation.

When constructing a classification model, the experimental validated pre-miRNAs (real pre-miRNAs) are used as positive samples. As nearly all reported miRNAs located in the untranslated regions or intergenic regions, the sequences with stem-loop structure (pseudo pre-miRNAs) are collected from the protein-coding regions. These pseudo pre-miRNAs are regarded as negative samples. In reality, the number of negative samples is far greater than positive samples, which forms the severe class imbalance problem. It is well studied that a classification model trained with these samples would tend to determine a new sequence as a pseudo pre-miRNA (the majority class) [15]. Simultaneously, it would result in poor classification with respect to the minority class (real pre-miRNAs).

Recently, several pre-miRNA classification methods have taken the imbalance problem into account. Triple-SVM [8] adopted random undersampling method to select the same number of negative samples with that of positive samples. PlantMiRNAPred [16] clustered the positive and negative samples according to their distribution. The equal number of representative positive and negative samples was selected as the training data. HuntMi [17] selected the partial negative samples based on the ROC score. Actually, there are a lot of various pseudo pre-miRNAs (negative samples) in the genomes. However, the methods with undersampling strategies ignored so much information of negative samples that the classification model has poor discriminative ability on them. In addition, miRNApre [18] only extracted the negative samples which are similar to the positive samples. As a result, the corresponding classification model has low robustness especially for the new pre-miRNAs that are not similar to the previous pre-miRNAs. Furthermore, this method also suffered from the negative effect of ignoring plenty of negative samples. On the other hand, microPred [19] generated the simulative positive samples based on SMOTE [20] to balance the numbers of positive and negative samples. Nonetheless, the process of generating simulative samples introduced the noisy data, which decrease the classification accuracy especially for positive samples. Therefore, it is essential to develop a new method to efficiently classify the imbalanced real and pseudo pre-miRNAs.

In addition, the correct classification of real pre-miRNAs (positive samples) has greater value than the correct classification of pseudo pre-miRNAs (negative samples). It is helpful for the biologists to discover more novel pre-miRNAs. Hence a better classification model should provide a higher discrimination ability on the positive samples. In order to construct this type of model, cost-sensitive and ensemble learning [21, 22] has taken the various misclassification costs of different categories of samples and the class imbalance into account and achieved superior performance. Therefore, we proposed a classification method based on cost-sensitive and ensemble learning, which completely exploits the class imbalance factor and the cost factor of different categories. Through a series of iterations, the samples that are easy to be classified correctly and those that are easy to be misclassified are determined gradually and are assigned different weights. During each iteration, a new classification instance is trained by using the equal numbers of positive and negative samples. The weights of samples are updated according to the classification result of the new instance. After the iteration process is finished, all the instances are integrated into an ensemble classifier.

First, each classification instance is trained by the same amount of positive and negative samples, which can efficiently relieve the negative effect of class imbalance. Second, the cost of misclassifying a positive sample is set higher than misclassifying a negative one. In this way, the classification accuracy on the positive samples is further improved. Third, the information of all the negative samples is exploited during the training process. At last, when a new classification instance is constructed, it focuses on the samples that are easy to be misclassified, which contributes to the improvement of the global classification performance. In addition, the data about human, animal, and plant were used to test our method, several state-of-the-art methods, and some classification models. Our method achieved superior classification performance not only for the cross validation but also for the testing on the newly added pre-miRNAs.

The rest of this paper is organized as follows.  Section 2 describes the features of real/pseudo pre-miRNAs and the process of constructing the ensemble classification model. In Section 3, we discuss the evaluation metrics of classification performance and compare and analyze the results of our method, other methods, and other classification models.

## 2. Methods

### 2.1. Features of Pre-miRNAs

It has been well studied that the pre-miRNAs have some characteristics about their sequences, secondary structures, and energy [23, 24]. The sequences and secondary structure of pre-miRNAs are usually conserved. The secondary structures often contain stem and loop regions. Furthermore, they have lower free energy. The previous pre-miRNA classification methods usually extracted different feature sets, as they focused on different characteristics of pre-miRNAs. So far, miPred [10], microPred [19], triplet-SVM [8], and miRNApre [18] have extracted the sequence-related, structure-related, and energy-related features. All the features are summarized as follows.

First, there are 81 sequence-related features. (1) 16 features are related to the two adjacent nucleotides, and they represent the frequencies of two adjacent nucleotides, denoted as *XY*%. *X* and *Y* can be A (adenine), G (guanine), C (cytosine), or U (uracil). (2) The G + C% is used to describe the total content of G and C in the sequences. (3) 64 features are related to the frequencies of three adjacent nucleotides and denoted as *XYZ*%, where *X*, *Y*, and *Z* ∈ {A, G, C, U}.

The following 49 features are related to the secondary structures of pre-miRNAs which can be obtained by using the structure-related prediction software such as the Vienna software package RNAfold [25]. (1) There are 8 features about the structure topology, including the structural diversity property* Diversity*, the structural frequency property* Freq*, the structural entropy-related properties* dS* and* dS/L*, the structural enthalpy-related properties* dH* and* dH/L*, the Shannon entropy value* dQ*, and the compactness of topology* dF*. (2) There are 9 features about base pairs, including the average amounts of base pairs |A − U | /*L*, |G − C | /*L*, and |G − U | /*L*, the percentages of base pairs within the stem region |A − U | %/*n_stems*, |G − C | %/*n_stems*, and |G − U | %/*n_stems*, the average number of base pairs in the stem region* Avg_BP_Stem*, the distance between a pair of bases* dD*, and the base-pairing tendency* dP*. (3) There are 32 features related to the characteristic of three adjacent nucleotides. “(” and “.” represent paired and unpaired bases, respectively. The nucleotide at the middle is recorded as A, G, C, or U. Thus, the percentages of the 32 combinations can be obtained.

Finally, there are 9 features related to the energy of secondary structure: (1) the minimal free energy of the secondary structure* dG*, the minimal free energy-related features* MFEI*_*1*_,* MFEI*_*2*_,* MFEI*_*3*_, and* MFEI*_*4*_, and the overall free energy* NEFE*, the energy combination features *Diff* = |*MFE* − *EFE* | /*L*, and (2) the energy required for dissolving the secondary structure *T*_*m*_ and *T*_*m*_/*L*.

These features have been successfully used to classify the real and pseudo pre-miRNAs. Therefore, the total of 139 features were merged to form our feature set. During the process of our feature extraction, the codes of miPred, microPred, triplet-SVM, and miRNApre were executed to obtain the respective features. All the extracted features were merged to represent each of real and pseudo pre-miRNAs.

### 2.2. Constructing the Cost-Sensitive Ensemble Model

**Figure 1:**
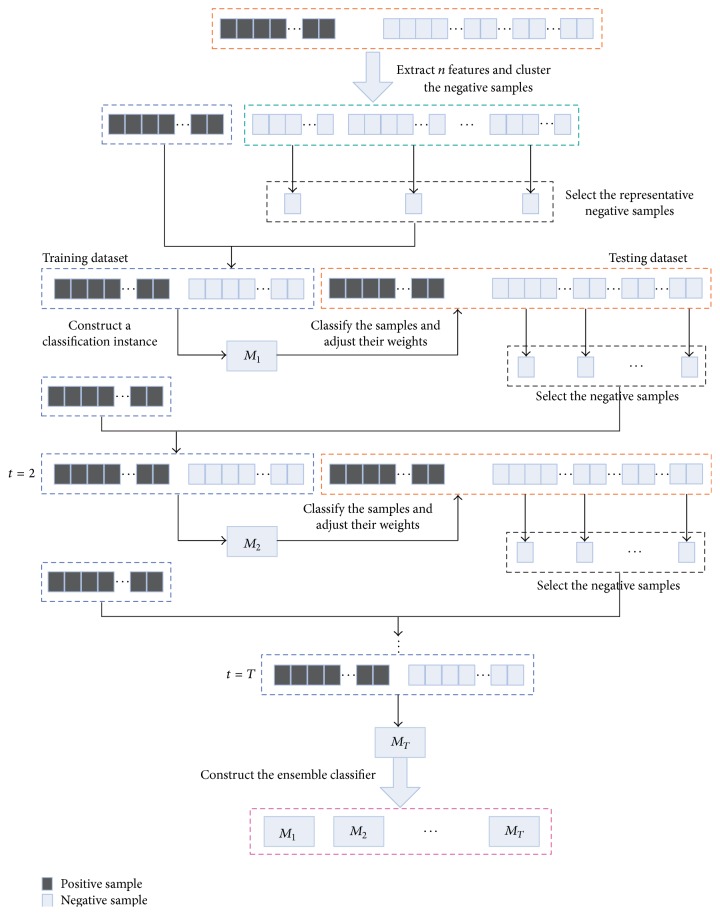
Constructing the integrated model to classify the real/pseudo pre-miRNAs.

A pre-miRNA classification method based on cost-sensitive ensemble learning is proposed and referred to as MiRNAClassify. We constructed multiple classification instances through a series of iterations and integrated these instances into an ensemble classifier. In this way, the information of all the positive and negative samples can be exploited. In the process of each iteration, a new classification instance is trained by the equal number of positive and negative samples, which can protect it from the negative effect of class imbalance. At the same time, each sample has its own weight which reflects the degree that it is easy to be misclassified. The training samples are selected in proportion to their weights, which make the new classification instance focus on the samples that are easy to be misclassified. Moreover, the weights of samples are adjusted according to the classification result of each instance. Hence when constructing a new instance, the new weight distribution of samples can be integrated to obtain a more accurate instance. In addition, as the cost of misclassifying each positive sample is higher than each negative sample, the positive samples are assigned greater cost weights. As shown in Figure 1, constructing the ensemble classification model includes the following 4 steps.

(1) The sample set *V* contains all the positive samples (real pre-miRNAs) and the negative samples (pseudo pre-miRNAs). *n* is the number of the features. We extracted *n* features from each positive sample and each negative one. Since the purpose of our cost-sensitive learning is to improve the discrimination ability on the positive samples (small class), the cost weights of positive samples should be set higher than those of negative samples. *C*_*P*_ and *C*_*N*_ represent the misclassification cost of positive and negative samples, respectively. The weight of each positive sample is initially set to *C*_*P*_/|*V*| and that of each negative one is set to *C*_*N*_/|*V*| where |*V*| is the size of *V*. As *C*_*P*_ should be higher than *C*_*N*_, *C*_*P*_ was set as 1 and *C*_*N*_ varied from 0.1 to 0.9 with step length of 0.1. The best classification performance was achieved when *C*_*P*_ = 1 and *C*_*N*_ = 0.6.

During the process of the first iteration, in the *n*-dimensional sample space, the distance between any two of negative samples is calculated. Next, all the negative samples in *V* are gathered into *k* clusters based on *K*-means, and *k* equals the number of positive samples. For each cluster, the negative sample that is closest to the center is obtained to be a training sample. Thus the initial training dataset *A*_1_ is composed of *k* positive samples and *k* negative samples. The first classification instance based on the support vector machine (SVM) is trained by *A*_1_ and denoted as *M*_1_. The purpose of constructing *M*_1_ according to above steps is to quickly determine the positive and negative samples that are easy to be misclassified. As *M*_1_ is trained by the same number of positive and negative samples, *M*_1_ was not affected by the class imbalance.

It has been confirmed that Rogers and Tanimoto measurement [26] could successfully measure the similarity between pre-miRNAs [16]. On the basis of the similarity measurement, the distance between two negative samples, such as *x* and *y*, is defined as follows:

(1) $$\begin{array}{c} \text{dis}\left(\;v_{x},v_{y}\;\right)=1-\frac{v_{x}^{t}\cdot v_{y}}{v_{x}^{t}\cdot v_{x}+v_{y}^{t}\cdot v_{y}-v_{x}^{t}\cdot v_{y}}, \end{array}$$

where *v*_*x*_ and *v*_*y*_ are the feature vectors of *x* and *y*, respectively, and *v*_*x*_^*t*^ and *v*_*y*_^*t*^ represent their transpose.

(2) During the process of each subsequent iteration, the classification instance *M*_*t*_ (1 ≤ *t* ≤ *T*) classifies all the positive and negative samples in *V*. *G*_*m*_^*t*^ represents the global classification performance of *M*_*t*_ and it is described in detail in Section 3.2. The weight of each positive and negative sample is adjusted according to the classification results of *M*_*t*_. The weight of a sample that was classified correctly is reduced and that of a sample that was misclassified remains unchanged. After a series of iterations were completed, a greater weight represents that a sample has been misclassified for more times.

As *G*_*m*_^*t*^ is *M*_*t*_'s classification performance, the error rate of *M*_*t*_ is *φ*_*t*_ = 1 −  *G*_*m*_^*t*^. *θ*_*t*_ is a parameter for adjusting the weight of each sample and *θ*_*t*_ = *φ*_*t*_/(1 − *φ*_*t*_). Thus, the weight of the *i*th sample *x*_*i*_ at the *t*th iteration is updated as *w*_*i*_^*t*−1^ *θ*_*t*_^1−|*p*_*t*_(*x*_*i*_) − *y*_*i*_|^. Here, *w*_*i*_^*t*−1^ is the weight of *x*_*i*_ at the (*t* − 1)th iteration, and *y*_*i*_ is the true label of *x*_*i*_. If *x*_*i*_ is a real pre-miRNA, *y*_*i*_ is 1. Otherwise, *x*_*i*_ is a pseudo pre-miRNA and *y*_*i*_ is 0. *p*_*t*_(*x*_*i*_) represents the classification result of *M*_*t*_ over *x*_*i*_, and 1 and 0 represent that *x*_*i*_ is classified as positive sample and negative one, respectively. If *x*_*i*_ is classified correctly, the value of *p*_*t*_(*x*_*i*_) is the same as its true label *y*_*i*_, we have |*p*_*t*_(*x*_*i*_) − *y*_*i*_ | = 0 and 1 − |*p*_*t*_(*x*_*i*_) − *y*_*i*_ | = 1. The weights of the correctly classified samples are multiplied by *θ*_*t*_ which is smaller than 1. Moreover, if the classification error rate is lower, the weights of the samples will become smaller. In terms of the misclassified samples, we have |*p*_*t*_(*x*_*i*_) − *y*_*i*_ | = 1 and 1 − |*p*_*t*_(*x*_*i*_) − *y*_*i*_ | = 0. Thus, their weights remain unchanged.

(3) Assume the number of positive samples is *k*. To make the balance of positive and negative samples, we also selected *k* negative samples. A negative sample with greater weight means that it has been misclassified for more times in the previous iterations. Therefore, we selected the negative samples in proportion to their weights. A new classification instance *M*_*t*+1_ is trained by *k* negative samples and *k* positive samples. At the same time, the weights of the training samples are integrated.

(4) Steps (2) and (3) are repeatedly performed until the termination condition is satisfied.

At last, *T* classification instances are constructed and denoted as *M*_1_, *M*_2_,…, *M*_*T*_. The results of all instances are integrated based on the voting mechanism to give the final classification result. If the classification error rate *φ*_*t*_ is lower, *θ*_*t*_ is smaller and the determination weight log⁡(1/*θ*_*t*_) is greater. In this way, the result given by a instance with higher classification performance accounts for the greater proportion for the final result during the voting process. The algorithm of classification of the real/pseudo pre-miRNAs based on cost-sensitive ensemble learning is illustrated in Algorithm 1.

The iterative process is terminated after the while loop is performed for *T* times. When the value of *T* is large enough, the error rate of the ensemble classifier can reach values as small as possible. In this study, the value of *T* is set as 300.

## 3. Results and Discussion

### 3.1. Data Preparation

Both the sample set and the feature set are important factors influencing the pre-miRNA classification. Furthermore, the previous methods extracted various features because they focused on the different characteristics of pre-miRNAs. Therefore, in order to fairly compare with each of previous methods, our classification method was also trained by the sample set and feature set which were the same as its ones. The information about the sample and feature of each compared method is listed as follows.

MicroPred collected 691 real pre-miRNAs from the early version of miRNA database miRBase [27] (12.0 version, abbreviated as miRBase12.0) as the positive samples. The 660 pre-miRNAs have the stem-loop secondary structures and 31 pre-miRNAs have multiple stem-loops. These positive samples formed a positive dataset which is referred to as “MP_positive_set.” MicroPred obtained the 8494 pseudo hairpins as the negative samples and these hairpins were extracted from the human protein-coding regions. In addition, microPred collected 754 other types of noncoding RNAs, such as tRNA and rRNA, as the negative samples. The total 9248 negative samples formed a negative dataset named “MP_negative_set.” In terms of feature set, microPred obtained 21 features to train its classification model.

PlantMiRNAPred was a classical method used to classify the plant pre-miRNAs. It collected the 2043 plant pre-miRNAs in the miRBase14.0 as the positive samples and extracted the 2122 pseudo hairpins from the protein-coding regions of* Arabidopsis thaliana* and* Glycine max* as the negative samples. The positive samples and negative samples of PlantMiRNAPred formed its positive dataset “PMP_positive_set” and negative one “PMP_negative_set.” PlantMiRNAPred extracted 68 features from plant pre-miRNAs.

HuntMi obtained 1406 human pre-miRNAs from miRBase17.0 and extracted 81228 human pseudo hairpins to form the human positive dataset “HM_hsa_positive_set” and negative one “HM_hsa_negative_set.” It also obtained 7053 real animal pre-miRNAs and 218154 animal pseudo hairpins to construct the animal positive dataset “HM_animal_positive_set” and negative one “HM_animal_negative_set,” respectively. In addition, it obtained 2172 plant pre-miRNAs and 114929 plant pseudo hairpins to form the plant positive dataset “HM_plant_positive_set” and negative one “HM_plant_negative_set.” HuntMi used 28 features to train its model.

MiRNApre obtained 1496 human pre-miRNAs from miRBase17.0 as the positive samples and extracted 1446 pseudo hairpins similar with the positive samples. These pseudo hairpins are used as the negative samples. These positive samples and negative samples formed the positive and negative datasets “MP_positive_set” and “MP_negative_set.” MiRNApre extracted 98 features from each positive and negative sample.

Moreover, the new pre-miRNAs about human,* Arabidopsis lyrata*,* Oryza sativa*, and* Glycine max* were reported by miRBase when our work was almost completed. These pre-miRNAs were also used to test the classification methods, which can further validate their ability of discovering the new pre-miRNAs.

In addition, to compare our method with other methods simultaneously, all the positive and negative samples about human, animal, and plant were merged, respectively. The 1496 human pre-miRNAs and 81982 pseudo hairpins formed the human-related positive and negative datasets which are referred to as “Merged_hsa_positive_set” and “Merged_hsa_negative_set.” The animal-related positive and negative datasets contain 7053 real animal pre-miRNAs and 218154 pseudo hairpins and they are named “Merged_animal_positive_set” and “Merged_animal_negative_set.” The plant-related datasets are “Merged_plant_positive_set” and “Merged_plant_negative_set” which are composed of 2172 plant real pre-miRNAs and 117051 pseudo hairpins. All the classification methods were tested by performing 5-fold cross validation on the three groups of datasets. The 355 new human pre-miRNAs (updated human dataset), 68* Arabidopsis thaliana* pre-miRNAs (updated ath dataset), 169* Oryza sativa* pre-miRNAs (updated osa dataset), and 302* Glycine max* pre-miRNAs (updated gma dataset) were also used for testing. In terms of features, the 139 features described in Section 2.1 are used to represent each positive sample and negative one.

### 3.2. Performance Evaluation Metrics

Suppose that TP and TN represent the number of the correctly classified positive samples (real pre-miRNAs) and that of the correctly classified negative samples (pseudo pre-miRNAs), respectively. FP and FN represent the numbers of the misclassified positive and negative samples, respectively. Sensitivity (SE) represents the proportion of the positive samples that are classified successfully accounting for the total positive samples. Specificity (SP) represents the proportion of the successfully classified negative samples accounting for the total negative samples. Consider

(2) SE=TPTP+FN,SP=TNTN+FP.

For the pre-miRNA classification, if a classifier has higher SE and lower SP, it has poorer ability to identify the pseudo pre-miRNAs. Thus, many pseudo pre-miRNAs will be misclassified as the real pre-miRNAs, which reduces the possibility that the biological experiments can successfully identify pre-miRNAs. On the contrary, the classifier has poorer ability to identify the real pre-miRNAs, and many real pre-miRNAs will be misclassified as the pseudo pre-miRNAs, which reduces the possibility that the real pre-miRNAs are discovered by the experimental study. Therefore, the classifier should have both high SE and high SP.

The global classification accuracy based on machine learning is usually evaluated by the parameter Acc. However, the number of pseudo pre-miRNAs is usually much greater than that of real pre-miRNAs, which causes TN and FP to be much higher than TP and FN. Then we have

(3) $$\begin{array}{c} \text{Acc}=\frac{\text{TP}+\text{TN}}{\text{TP}+\text{TN}+\text{FP}+\text{FN}}\approx \frac{\text{TN}}{\text{TN}+\text{FP}}=\text{SP}. \end{array}$$

Therefore, besides SE and SP, we also compute the geometric mean of SE and SP, denoted as *G*_*m*_, to evaluate the global classification performance:

(4) $$\begin{array}{c} G_{m}=\sqrt{\text{SE}\times \text{SP}}. \end{array}$$

### 3.3. Comparison with Other Methods and Classification Models

In order to compare MiRNAClassify with the state-of-the-art methods microPred [19], PlantMiRNAPred [16], HuntMi [17], and miRNApre [18], the 5-fold cross validation is performed. During the process of the cross validation, the positive and negative samples are randomly divided into 5 parts. 4 parts are used as the training samples, and the remaining part is used for testing. The testing dataset does not intersect with the training dataset. So it can objectively test the classification performances of the methods. Besides cross validation, the newly added human, animal, and plant pre-miRNAs are used to test the ability of discovering the new pre-miRNAs.

MiRNAClassify is compared with other methods in two different ways. For one thing, in terms of each compared method, we choose its sample set and feature set to train MiRNAClassify. In this way, we compare MiRNAClassify with other methods, respectively. For another, MiRNAClassify is compared with all the other methods simultaneously by using the merged datasets and our feature set. In addition, MiRNAClassify is compared with other classification models, including SVM, naive Bayes, and Random Forest.

#### 3.3.1. Comparison Using the Same Dataset and Feature Set

**Figure 2:**
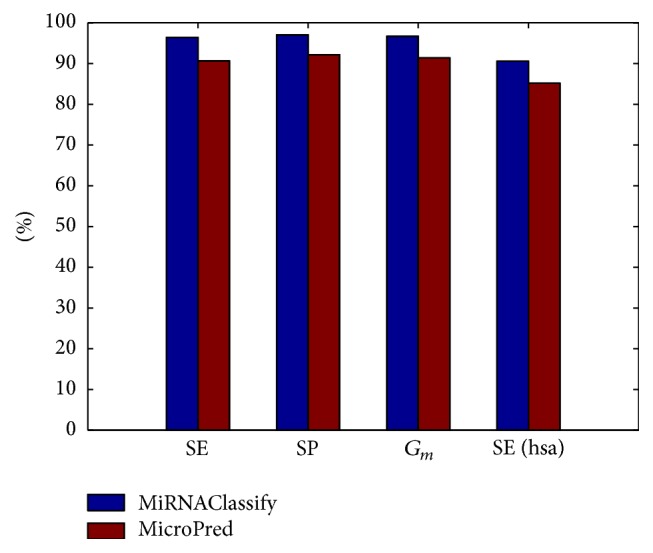
Comparison of the performances of MiRNAClassify and microPred.

MicroPred focused on classification of the human pre-miRNAs, and it generated new simulated positive samples based on SMOTE. In order to compare with microPred in a fair way, we used the sample set and feature set of microPred to train the model of MiRNAClassify. As shown in Figure 2 (details in Table 1), the first 3 columns are the results based on 5-fold cross validation, and the last column is the result for testing the newly added human pre-miRNAs. MicroPred obtained the lower sensitivity (SE = 90.65%) and specificity (SP = 92.16%) in the cross validation. The possible reason is that generating samples based on SMOTE introduced the noise data. MiRNAClassify is 5.31% better than microPred in overall accuracy *G*_*m*_. SE increased by 5.75% and SP increased by 4.87%. For the dataset that composed of 1186 newly added human pre-miRNAs, MP_updated_set, the SEs of MiRNAClassify and microPred are 90.56% and 85.16%, respectively. These SEs are not as good as those in the cross validation. The main reason is that MicroPred used the pre-miRNAs in the early version of miRBase and the new pre-miRNAs for testing are even more than the pre-miRNAs used to train its classification model.

**Figure 3:**
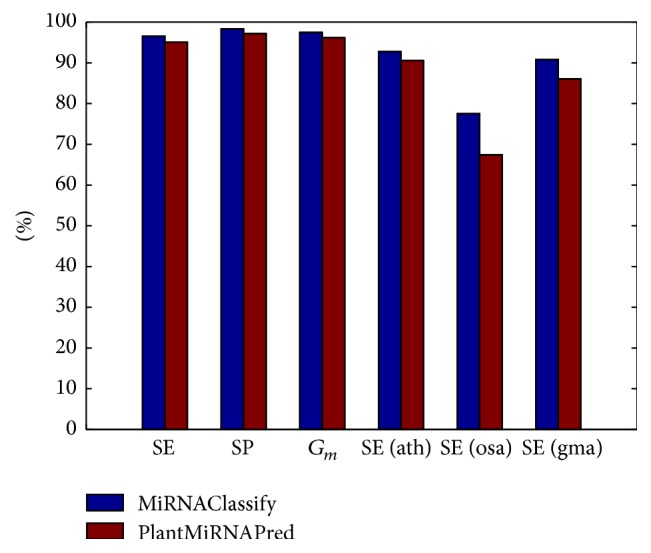
Comparison of the performances of MiRNAClassify and PlantMiRNAPred.

PlantMiRNAPred mainly studied the classification of plant pre-miRNAs. The model of MiRNAClassify was constructed by using the same sample and feature sets with PlantMiRNAPred. As shown in Figure 3 (details in Table 2), the first 3 columns are the comparison results of cross validation. MiRNAClassify achieved slightly better performance than PlantMiRNAPred. PlantMiRNAPred only extracted a small number of pseudo hairpins. The number of negative samples is close to that of positive samples, and there is no obvious class imbalance. It confirms that MiRNAClassify is still effective for this type of data. Actually, PlantMiRNAPred only selected partial positive and negative samples to train their model, which results in some of the sample information was lost. On the contrary, MiRNAClassify fully exploited the information from all the positive and negative samples by adjusting the sample weights and constructing multiple classification instances. In Figure 3, the last 3 columns are the results on 138 newly added* Arabidopsis thaliana* pre-miRNAs, 178* Oryza sativa* pre-miRNAs, and 488* Glycine max* pre-miRNAs. MiRNAClassify obtained consistently better classification accuracies.

**Figure 4:**
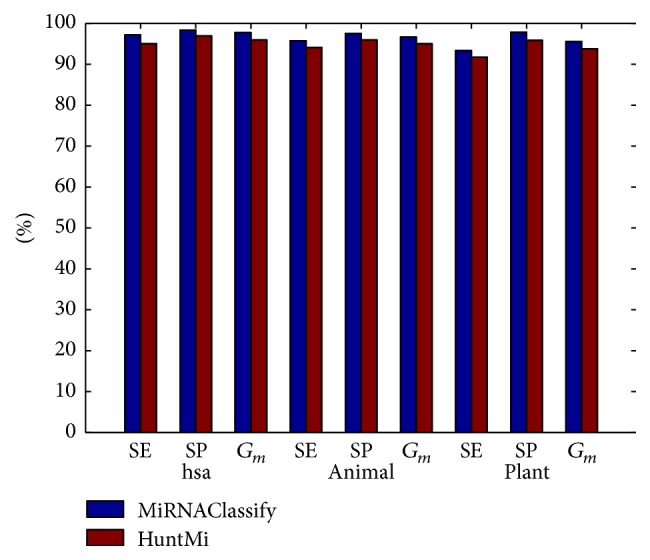
Comparison of the performances of MiRNAClassify and HuntMi based on 5-fold cross validation.

**Figure 5:**
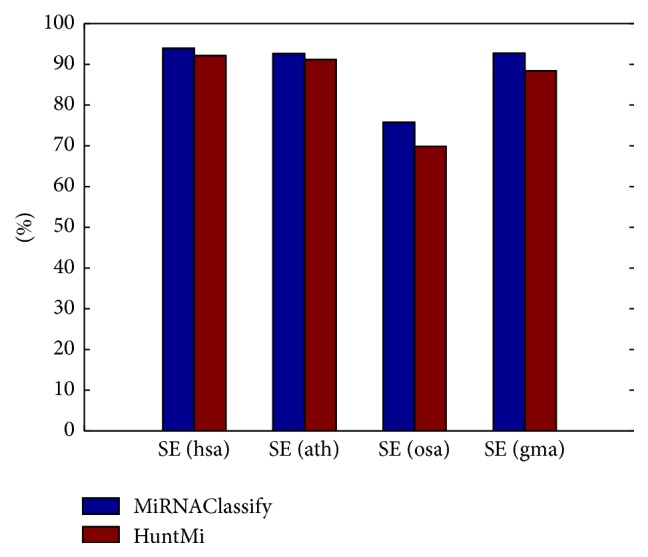
Comparison of the performances of MiRNAClassify and HuntMi on the newly added data.

HuntMi was constructed to distinguish the real and pseudo pre-miRNAs about human, animal, and plant. The model of MiRNAClassify was also trained by using its samples and features. As Figure 4 shows (details in Table 3), MiRNAClassify and HuntMi performed the cross validation on three groups of datasets, respectively. It is obvious that the number of negative samples is far more than that of positive samples. There is a severe imbalance between the positive samples and negative samples. Although there are quite a lot of negative samples, HuntMi selected a small number of negative samples based on the ROC values to train its model. Apparently, it lost a large amount of information of negative samples. MiRNAClassify completely presented its advantage in processing the class imbalance and achieved superior performance. In addition, we performed further testing using the newly added human,* Arabidopsis thaliana*,* Oryza sativa*, and* Glycine max* pre-miRNAs. The result was demonstrated in Figure 5, which confirms that MiRNAClassify can discover more new pre-miRNAs. We found that the classification performances of these two methods on newly added plant pre-miRNAs were worse than the performances on the new human pre-miRNAs. One of the important reasons is that the sequences and structures of plant pre-miRNAs are more complicated than those of human pre-miRNAs.

**Figure 6:**
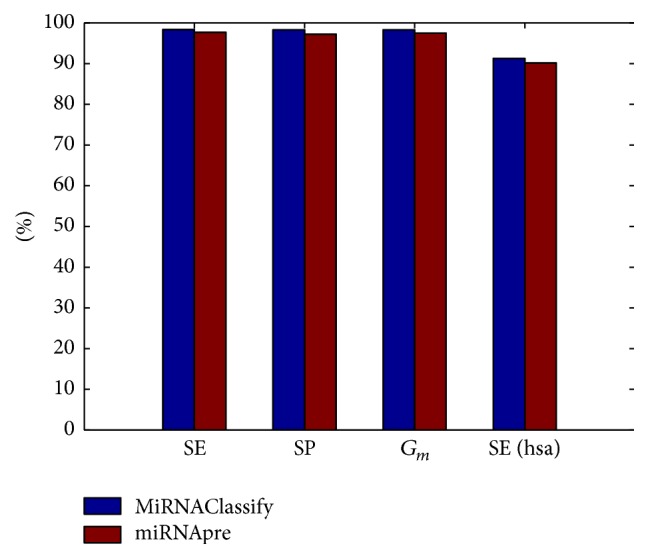
Comparison of the performances of MiRNAClassify and miRNApre.

Although miRNApre was tested on human, animal, and plant, only the training and testing samples about human could be obtained. Therefore, the human-related dataset was used to train the model of MiRNAClassify. Since miRNApre only selected the negative samples which are similar to the positive ones, the negative dataset is only composed of 1446 samples. The positive dataset contains 1496 samples (Table 4). There is no class imbalance because the number of positive samples nearly equals that of negative samples. In this case, the cross validation performance of MiRNAClassify is still better than miRNApre, as shown in the first 3 columns of Figure 6. In addition, we obtained 355 new human pre-miRNAs for further testing. The SEs of MiRNAClassify and miRNApre are 91.2% and 90.1%, respectively. The SEs are not as good as those obtained in the cross validation. The reason may be that the extracted negative samples were very similar to the positive samples which resulted in the low robustness of the classification models.

#### 3.3.2. Comparison over the Merged Datasets

Besides comparison with each method, respectively, we tested MiRNAClassify and other methods simultaneously by using the merged datasets about human, animal, and plant and the newly updated datasets and extracting a set of same features. It is worth noting that the number of negative samples is much greater than that of positive samples in each merged dataset. The classification results are demonstrated in Table 5.

MiRNAClassify performed the best not only over the merged datasets but also over the updated datasets. HuntMiRNA also achieved decent prediction performance. MicroPred, PlantMiRNAPred, and miRNApre obtained the inferior results, especially over the negative samples. In terms of MicroPred, more simulated positive samples have to be generated because there are so many negative samples, which also introduced more noisy data. The possible reason for the worse performance of PlantMiRNAPred and miRNApre is that most of information about negative samples was abandoned and their discriminative ability on negative samples was reduced greatly.

In addition, a paired *t*-test was used to determine whether MiRNAClassify's global performance (*G*_*m*_) over the 3 groups of merged datasets and its accuracy (SE) over the 4 updated datasets is higher than other methods. The corresponding *p* values are listed in Table 6. The statistic results confirm that MiRNAClassify outperforms other methods significantly at the significance level 0.05.

#### 3.3.3. Comparison with Other Classification Models

We use 5-fold cross validation to compare MiRNAClassify with other well-known classification models including the standard SVM, naive Bayes, and Random Forest. These models under SMOTE method were further developed to compare with MiRNAClassify. All the models were trained by the merged datasets and the same set of features, and their classification results are demonstrated in Table 7.

For the datasets with severe class imbalance, MiRNAClassify demonstrated its ability to process the imbalanced data and achieved the best performance. As expected, the standard classification models overlearned the information of majority class and obtained the small SEs and the great SPs. After the SMOTE method was applied to balance the positive and negative samples, these models except naive Bayes obtained decent improvement on *G*_*m*_ values. The main reason is that naive Bayes has intrinsic resistance to the class imbalance. However, the classification models under SMOTE method, SVM, and Random Forest obtained nearly consistent performances which were slightly better than naive Bayes. It confirmed that SVM has the excellent generalization ability. In particular, each classification instance of MiRNAClassify was established based on SVM, which was one of the important factors that MiRNAClassify could perform well. After exerting *t*-test on the *G*_*m*_ values obtained by MiRNAClassify and other models, the statistic results in Table 8 indicated that MiRNAClassify achieved significantly better performance.

## 4. Conclusions

A new method based on cost-sensitive and ensemble learning (MiRNAClassify) was developed to classify the imbalanced real and pseudo pre-miRNAs. The multiple classification instances were constructed and integrated to classify a query sequence. Each instance was trained by the same number of positive and negative samples, which effectively relieved the negative effect of class imbalance. At the same time, the information of all the positive and negative samples was completely exploited. The weight of each sample embodied the possibility that it would be misclassified. Based on the sample weight, the new classification instance could focus on the samples that are easy to be misclassified. All of the above contribute to the more accurate classification performance.

MiRNAClassify has been compared with the previous methods, microPred, PlantMiRNAPred, HuntMi, and miRNApre. Not only the human data but also the animal and plant data were used to test their performance. MiRNAClassify outperformed better than other methods during the cross validation and could discover more newly added pre-miRNAs. In addition, we compared MiRNAClassify with other methods and several well-known classification models by using the merged and imbalanced datasets. MiRNAClassify demonstrated its ability of processing the imbalance and achieved significantly better performance.

## Figures and Tables

**Algorithm 1:**
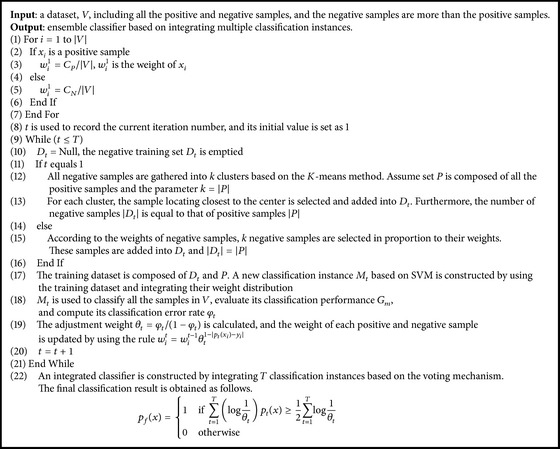
Algorithm of classifying the real/pseudo pre-miRNAs based on cost-sensitive ensemble learning.

**Table 1:** Datasets and detailed classification results of MiRNAClassify and microPred.

Method	Species	Dataset	Type	Size	SE (%)	SP (%)	*G*_*m*_ (%)
MiRNAClassify	*Homo sapiens*	MP_positive_set	Real	691	96.40	97.03	96.71
microPred		MP_negative_set	Pseudo	9248	90.65	92.16	91.40
MiRNAClassify		MP_updated_set	Real	1186	90.56		
microPred					85.16		

**Table 2:** Datasets and detailed classification results of MiRNAClassify and PlantMiRNAPred.

Method	Species	Dataset	Type	Size	SE (%)	SP (%)	*G*_*m*_ (%)
MiRNAClassify	*Plant*	PMP_positive_set	Real	2043	96.57	98.35	97.46
PlantMiRNAPred		PMP_negative_set	Pseudo	2122	95.10	97.17	96.13

MiRNAClassify	*Arabidopsis thaliana*	PMP_ath_updated_set	Real	138	92.75		
PlantMiRNAPred					90.58		

MiRNAClassify	*Oryza sativa*	PMP_osa_updated_set	Real	178	77.53		
PlantMiRNAPred					67.42		

MiRNAClassify	*Glycine max*	PMP_gma_updated_set	Real	488	90.78		
PlantMiRNAPred					86.07		

**Table 3:** Datasets and detailed classification results of MiRNAClassify and HuntMi.

Method	Species	Dataset	Type	Size	SE (%)	SP (%)	*G*_*m*_ (%)
MiRNAClassify	*Homo sapiens*	HM_hsa_positive_set	Real	1406	97.15	98.35	97.75
HuntMi		HM_hsa_negative_set	Pseudo	81228	95.02	96.94	95.98

MiRNAClassify	Animal	HM_animal_positive_set	Real	7053	95.74	97.55	96.64
HuntMi		HM_animal_negative_set	Pseudo	218154	94.11	95.95	95.03

MiRNAClassify	Plant	HM_plant_positive_set	Real	2172	93.32	97.82	95.54
HuntMi		HM_plant_negative_set	Pseudo	114929	91.71	95.87	93.77

MiRNAClassify	*Homo sapiens*	HM_hsa_updated_set	Real	445	93.93		
HuntMi					92.14		

MiRNAClassify	*Arabidopsis thaliana*	HM_ath_updated_set	Real	68	92.65		
HuntMi					91.18		

MiRNAClassify	*Oryza sativa*	HM_osa_updated_set	Real	169	75.74		
HuntMi					69.82		

MiRNAClassify	*Glycine max*	HM_gma_updated_set	Real	302	92.72		
HuntMi					88.41		

**Table 4:** Datasets and detailed classification results of MiRNAClassify and miRNApre.

Method	Species	Dataset	Type	Size	SE (%)	SP (%)	*G*_*m*_ (%)
MiRNAClassify	*Homo sapiens*	MP_positive_set	Real	1496	98.33	98.27	98.29
miRNApre		MP_negative_set	Pseudo	1446	97.66	97.23	97.44
MiRNAClassify		MP_updated_set	Real	355	91.27		
miRNApre					90.14		

**Table 5:** The classification results of MiRNAClassify and other methods over the merged datasets and the updated datasets.

Method	Accuracy (%)	Merged human dataset	Merged animal dataset	Merged plant dataset	Updated human dataset	Updated ath dataset	Updated osa dataset	Updated gma dataset
MiRNAClassify	SE	97.93	95.85	93.37				
	SP	98.30	97.62	97.91	94.08	92.65	79.29	93.05
	*G*_*m*_	98.11	96.73	95.61				

MicroPred	SE	92.25	91.61	89.50				
	SP	95.70	94.85	93.10	91.27	89.71	66.86	85.76
	*G*_*m*_	93.96	93.21	91.28				

PlantMiRNAPred	SE	93.58	92.70	91.39				
	SP	91.20	88.60	87.10	92.11	89.71	70.42	88.41
	*G*_*m*_	92.38	90.63	89.22				

HuntMi	SE	95.32	94.14	91.76				
	SP	97.11	96.07	95.94	92.68	91.18	72.78	89.07
	*G*_*m*_	96.21	95.10	93.83				

miRNApre	SE	97.39	93.49	91.71				
	SP	90.90	89.80	88.10	90.14	89.71	71.01	86.09
	*G*_*m*_	94.09	91.63	89.89				

**Table 6:** The statistic results obtained by using paired *t*-test over the prediction performance of MiRNAClassify and that of another method.

Different datasets	microPred	PlantMiRNAPred	HuntMi	miRNApre
*p* values on three groups of datasets	0.0019	4.9374*e* − 04	9.7070*e* − 04	0.0050
*p* values on four updated datasets	0.0339	0.0284	0.0354	0.0108

**Table 7:** The classification results of MiRNAClassify and three classification models over the merged datasets.

Classification models	SE (%)	SP (%)	*G*_*m*_ (%)
Human			
SVM	69.18	99.83	83.11
SVM + SMOTE	92.25	95.70	93.96
Naive Bayes	87.43	96.12	91.67
Naive Bayes + SMOTE	90.24	94.43	92.31
Random Forest	67.78	99.82	82.26
Random Forest + SMOTE	91.51	95.34	93.41
MiRNAClassify	97.93	98.30	98.11

Animal			
SVM	69.03	98.14	82.31
SVM + SMOTE	91.61	94.85	93.21
Naive Bayes	85.04	95.03	89.90
Naive Bayes + SMOTE	90.83	92.61	91.71
Random Forest	69.52	98.72	82.84
Random Forest + SMOTE	91.12	95.01	93.05
MiRNAClassify	95.85	97.62	96.73

Plant			
SVM	68.51	99.24	82.45
SVM + SMOTE	89.50	93.10	91.28
Naive Bayes	82.91	96.75	89.57
Naive Bayes + SMOTE	87.20	92.61	89.86
Random Forest	68.32	99.35	82.39
Random Forest + SMOTE	89.18	92.87	91.01
MiRNAClassify	93.37	97.91	95.61

**Table 8:** The statistic results obtained by using paired *t*-test over the prediction performance of MiRNAClassify and that of another classification model.

*p* value	SVM	Naive Bayes	Random Forest
	7.3053*e* − 04	6.2651*e* − 04	0.0015

*p* value	SVM + SMOTE	Naive Bayes + SMOTE	Random Forest + SMOTE
	0.0019	0.0010	0.0028
